# Low-VAF TP53-Mutated AML Displays Distinct Biological Features in a Single-Center Cohort

**DOI:** 10.3390/biomedicines14010180

**Published:** 2026-01-14

**Authors:** Xiaoxuan Lu, Xiaohang Ma, Kainan Zhang, Shun Zhang, Fangfang Wei, Hao Jiang, Qian Jiang, Yingjun Chang, Xiaojun Huang, Xiaosu Zhao

**Affiliations:** 1 Beijing Key Laboratory of Cell and Gene Therapy for Hematologic Malignancies, Peking University People’s Hospital, Peking University Institute of Hematology, National Clinical Research Center for Hematologic Disease, Peking University, Beijing 100044, China; luxx1005@163.com (X.L.);; 2 Peking-Tsinghua Center for Life Sciences, Academy for Advanced Interdisciplinary Studies, Peking University, Beijing 100871, China; 3 Research Unit of Key Technique for Diagnosis and Treatments of Hematologic Malignancies, Chinese Academy of Medical Sciences, 2019RU029, Beijing 100730, China; 4 Collaborative Innovation Center of Hematology, Peking University, Beijing 100044, China

**Keywords:** AML, TP53 mutation, low-VAF

## Abstract

**Background**: The International Consensus Classification (ICC) currently proposes an empirical variant allele frequency (VAF) threshold of 10% to define TP53-mutated acute myeloid leukemia (AML) and to distinguish oncogenic driver from concomitant mutations. However, the optimal cutoff remains uncertain, and the biological and clinical features of low-VAF cases have not been systematically characterized. **Methods**: In this single-center retrospective cohort study, we stratified TP53-mutated AML by a 10% VAF cutoff and compared clinical characteristics, cytogenetic and molecular profiles, and survival outcomes between groups. **Results**: The VAF < 10% group exhibited a distinctive profile: fewer adverse cytogenetic abnormalities [complex karyotype, −7, −5/del(5q)], a more adverse molecular profile (EVI1 overexpression, greater co-mutation burden, higher frequencies of ASXL1 and SRSF2 mutations), and a higher proportion of CD34^+^CD38^−^ blast immunophenotype. TP53 hotspot mutations were also more common. Survival analyses showed poor prognosis in both groups, and the VAF < 10% group showed numerically longer survival without statistical significance, indicating no clear survival advantage for low VAF. **Conclusions**: These data support the clinical relevance of the ICC 10% threshold. TP53-mutated AML with VAF < 10% may represent a biologically distinct subgroup. Further multicenter studies with larger cohorts are needed to validate and refine the VAF threshold for prognostic evaluation and individualized management.

## 1. Introduction

TP53 mutations occur in approximately 13% of de novo AML cases [1], with higher frequencies observed in therapy-related AML, secondary AML, and in elderly patients [2,3,4]. Previous studies have demonstrated strong associations between TP53 mutations, complex karyotypes, together with consistently adverse outcomes in AML [5,6,7]. Despite the advent of multiple novel therapeutic strategies, the overall outcomes for patients with TP53-mutated AML have not substantially improved, highlighting a substantial unmet clinical need [8,9].

TP53 VAF may help distinguish dominant leukemic clones from clonal hematopoiesis (CH), however no consensus cutoff has been established. To recognize the diagnostic distinction between leukemic driver mutations and concurrent mutations associated with CH, the ICC adopts a pragmatic 10% threshold and stipulates that TP53-mutated myeloid neoplasms require a TP53 VAF > 10% [10]. However, the evidentiary basis for this threshold remains limited, and whether VAF alone can distinguish a dominant clone from CH remains controversial. Several studies also suggest that higher TP53 VAF cutoffs may be informative. Bahaj et al. observed that, among monoallelic TP53 mutations, VAF > 23% predicted poorer outcomes resembling those of biallelic inactivation [11], and other studies have examined a 20% threshold [12,13]. Results for patients with low VAF are likewise inconsistent. Grob et al., analyzing a cohort that included AML and MDS with excess blasts, found no association between overall survival and VAF across multiple bands (5%, 10%, 20%, 30%, 40%, 50%) [6]. By contrast, studies in MDS and secondary AML have linked VAF < 20% to more favorable outcomes [12], and a recent MDS/AML study has reported significantly worse survival in the AML subgroup when VAF exceeded 10% [14].

Given this context, we analyzed a single-center, real-world cohort of TP53-mutated AML and dichotomized patients by a 10% VAF to compare biological features and outcomes, aiming to delineate the clinical and genetic profile of low-VAF TP53-mutated AML. As a sensitivity analysis, we repeated the comparisons using a 20% threshold to test the robustness of our findings and to help identify an appropriate VAF cutoff.

## 2. Materials and Methods

### 2.1. Patients and Samples

**Figure 1:**
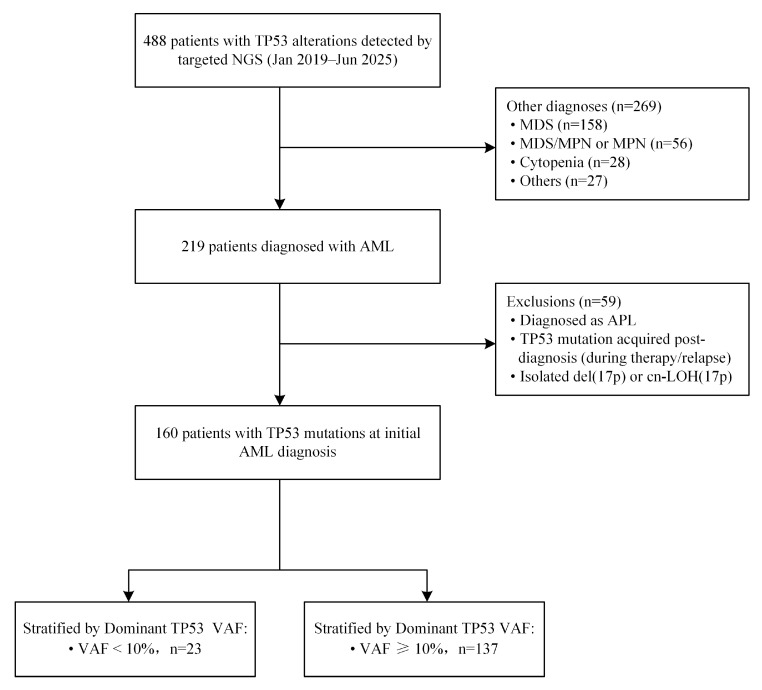
Flow diagram of cohort selection and VAF-based stratification in TP53-mutated AML. From 488 individuals with TP53 alterations by targeted NGS, 219 were diagnosed with AML; after exclusions, 160 with TP53 mutations at initial AML diagnosis were included and stratified by dominant TP53 VAF (<10% vs. ≥10%). (Dominant TP53 VAF was defined as the highest TP53 VAF per patient).

We conducted a single-center retrospective study to characterize the biological features and clinical outcomes of adults (≥18 years) with TP53-mutated AML. Between January 2019 and June 2025, targeted next-generation sequencing (NGS) identified 488 individuals with TP53 alterations at our institution. Among them, 219 were diagnosed with AML according to WHO 2022 criteria [15]. We excluded 59 AML patients for the following reasons: acute promyelocytic leukemia (APL), post-diagnosis acquisition of a TP53 mutation (i.e., absent at AML diagnosis but detected during therapy or at relapse), or isolated 17p lesions [del(17p) or copy-neutral loss of heterozygosity, cn-LOH(17p)] without a TP53 variant. The final cohort comprised 160 patients with a TP53 mutation at initial AML diagnosis. For analyses, patients were stratified by dominant TP53 VAF into <10% (n = 23) and ≥10% (n = 137) groups. When multiple TP53 variants were present, the highest VAF (dominant TP53) per patient was used for grouping (Figure 1).

### 2.2. Treatment

Induction chemotherapy regimens were administered in accordance with the Chinese guidelines for the diagnosis and treatment of adult acute myeloid leukemia [16]. Induction therapy consisted of intensive chemotherapy, including idarubicin–cytarabine (IA), daunorubicin–cytarabine (DA), and homoharringtonine–cytarabine–aclacinomycin (HAA) regimens. For patients who were not suitable candidates for in-tensive chemotherapy, lower-intensity treatment strategies such as HMA in combination with VEN were utilized.

After achieving CR/CRi, allo-HSCT was considered for patients with an available suitable donor [17]. While awaiting transplantation, consolidation therapy typically consisted of at least two cycles of intermediate-dose cytarabine (Ara-C)–based regimens.

Conditioning regimens, donor selection, HLA typing, stem cell collection, and graft-versus-host disease (GVHD) prophylaxis and management were performed ac-cording to previously reported methodologies [18,19].

### 2.3. TP53 Mutation Detection by Targeted NGS

Targeted NGS was performed at initial diagnosis using a 139-gene myeloid panel (Table S1). The TP53 allelic status was comprehensively evaluated by conventional karyotyping, fluorescence in situ hybridization, and copy number variation analyses. Patients were required to have at least one TP53 mutation to be included. Under high-coverage Illumina targeted sequencing with standard QC, the analytical sensitivity for detecting SNVs/short indels is approximately 0.5–1% VAF, consistent with prior reports [20]. Accordingly, variants with a VAF < 1% were excluded from further analysis.

### 2.4. Statistical Analysis

Overall survival (OS) and event-free survival (EFS) were defined according to the European LeukemiaNet (ELN2022) [21]. Categorical variables were presented as counts (percentages) and compared using Fisher’s exact test. Continuous variables were summarized using medians (ranges) and compared using the Mann–Whitney U test. Survival curves were estimated by the Kaplan–Meier method, and differences between groups were assessed using the log-rank test. Because this was a retrospective study, no a priori sample-size calculation was performed. A two-sided *p* value < 0.05 was considered statistically significant. We estimated the minimum detectable hazard ratio (MDHR) for the two-sided log-rank test, comparing patients with TP53 VAF < 10% versus ≥10%, assuming 80% power and a type I error rate (α) of 0.05, based on the observed number of events and the group allocation ratio. Analyses were performed using SPSS version 26.0 (IBM Corp., Armonk, NY, USA), and figures were generated with GraphPad Prism version 9.0 and R version 4.4.1.

## 3. Results

### 3.1. Patient Characteristics

A total of 160 patients with TP53-mutated AML were included in this retrospective study (patient selection, Figure 1). The median age was 61 years (range: 18–88), and 56.9% were male. Of these, 23 (14.4%) patients had TP53 VAF < 10% and 137 (85.6%) had VAF ≥ 10%. Age distribution (median: 63 vs. 61 years; *p* = 0.174) was comparable, and sex distribution did not differ significantly between groups (male: 73.9% vs. 54.0%; *p* = 0.110). Additionally, the VAF < 10% group had a higher proportion of secondary AML than the VAF ≥ 10% group (43.5% vs. 27.0%; *p* = 0.236) (Table 1).

Regarding hematologic parameters, patients with VAF < 10% had significantly lower white blood cell (WBC) counts compared with those with VAF ≥ 10% (median: 2.00 vs. 4.00 × 10^9^/L; *p* = 0.045). No significant differences were observed in hemoglobin levels (median: 88 vs. 74 g/L; *p* = 0.067) or platelet counts (median: 62.0 vs. 39.0 × 10^9^/L; *p* = 0.115) between the two groups.

With respect to blast percentage, the proportion of bone marrow blasts was similar between the groups (median: 36.0% vs. 39.8%; *p* = 0.223). However, peripheral blood blasts were significantly lower in the VAF < 10% group compared with the VAF ≥ 10% group (median: 2.0% vs. 22.5%; *p* < 0.001).

In addition, a comparison of blast immunophenotypes showed a significantly lower proportion of CD38^+^ cases in the VAF < 10% group (57.1% vs. 83.6%; *p* = 0.015) and a significantly higher proportion of the CD34^+^CD38^−^ leukemic stem cell (LSC) phenotype (38.1% vs. 15.7%; *p* = 0.030) (Figure S1), whereas no significant differences were observed between groups for CD34, CD117, CD33, CD13, HLA-DR, CD7, or CD123 expression (Table 1).

Data are N (%) unless otherwise indicated; continuous variables are reported as median (range). *p* values were calculated using Fisher’s exact test or the Wilcoxon rank-sum test, as appropriate. Abbreviations: AML, acute myeloid leukemia; VAF, variant allele frequency; WBC, white blood cell; Hb, hemoglobin; PLT, platelets; BM, bone marrow; PB, peripheral blood; sAML, secondary AML; t-AML, therapy-related AML.

### 3.2. Genetic Characterization

We compared cytogenetic features between the two groups. Compared with the VAF ≥ 10% group, patients with VAF < 10% had a markedly lower rate of complex karyotype (31.8% vs. 77.9%; *p* < 0.001). Among other established adverse-risk cytogenetic abnormalities, monosomy 7 (4.5% vs. 27.2%; *p* = 0.028) and monosomy 5/del(5q) (4.5% vs. 54.4%; *p* < 0.001) were significantly less frequent in the VAF < 10% group. 17p abnormalities (13.6% vs. 33.6%; *p* = 0.080) also tended to be less common in this group, but the differences were not statistically significant (Table 1).

**Figure 2:**
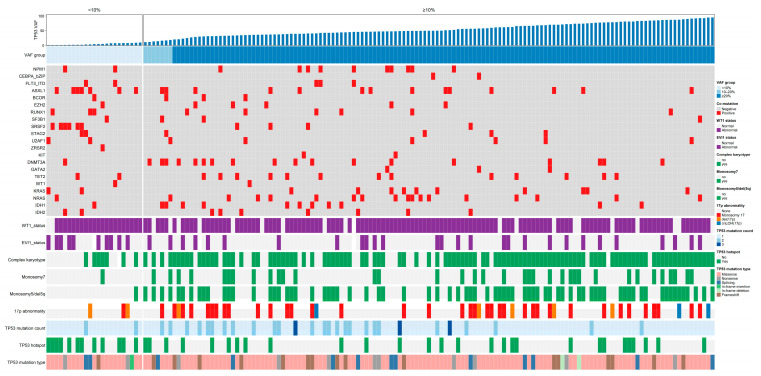
Landscape of cytogenetic and molecular features in TP53-mutated AML patients. Patients were ordered from left to right according to the variant allele frequency (VAF) of the dominant TP53 mutation at diagnosis. The oncoprint displays co-mutations in recurrently mutated genes, WT1 and EVI1 expression status, and key cytogenetic lesions including complex karyotype, monosomy 7, monosomy 5/del(5q), and 17p abnormalities [monosomy 17, del(17p), or cn-LOH(17p)]. TP53 mutation characteristics are also shown, including mutation count, hotspot status (R175, Y220, G245, R248, R273, R282), and mutation type (missense, nonsense, frameshift, splicing, in-frame insertion/deletion). Color codes are indicated in the legends.

To delineate the molecular landscape, we depicted the distribution of somatic co-mutations in an oncoprint (Figure 2), and the groupwise comparisons are summarized in Table 1. For gene expression, WT1 overexpression (threshold ≥ 0.6%) was prevalent in both groups (91.3% vs. 86.0%; *p* = 0.741), whereas EVI1 overexpression (threshold ≥ 8.0%) was more frequent in the VAF < 10% group (45.5% vs. 20.7%; *p* = 0.028).

All patients underwent targeted NGS testing at diagnosis using a 139-gene myeloid panel. Pathogenic/likely pathogenic variants were classified as Tier I–II and counted per patient. The VAF < 10% group carried a higher number of Tier I–II co-mutations than the VAF ≥ 10% group (median: 3 vs. 1; *p* = 0.001). At the single-gene level, mutations in ASXL1 (30.4% vs. 10.2%; *p* = 0.015) and SRSF2 (26.1% vs. 2.9%; *p* = 0.001) were significantly more frequent in the VAF < 10% group, whereas frequencies of other genes did not differ significantly between groups (Table 1).

### 3.3. TP53 Mutation Characteristics

The number of TP53 mutations did not differ significantly between the VAF < 10% and VAF ≥ 10% groups (median: 1 vs. 1; *p* = 0.177). The proportion of patients with multiple mutations (≥2) was also comparable (8.7% vs. 20.4%; *p* = 0.253).

Based on the dominant TP53 variant (highest VAF at diagnosis), 26.1% (6/23) of patients in the VAF < 10% group had VAF < 2%, 26.1% (6/23) had 2% ≤ VAF < 5%, and 47.8% (11/23) had 5% ≤ VAF < 10%. In the VAF ≥ 10% group, 5.1% (7/137) had 10% ≤ VAF < 20%, 46.0% (63/137) had 20% ≤ VAF < 50%, and 48.9% (67/137) had VAF ≥ 50%. According to ICC, the allelic status in the VAF ≥ 10% group included 17.5% (24/137) single-hit, 70.1% (96/137) multi-hit, and 12.4% (17/137) multi-hit equivalent.

**Figure 3:**
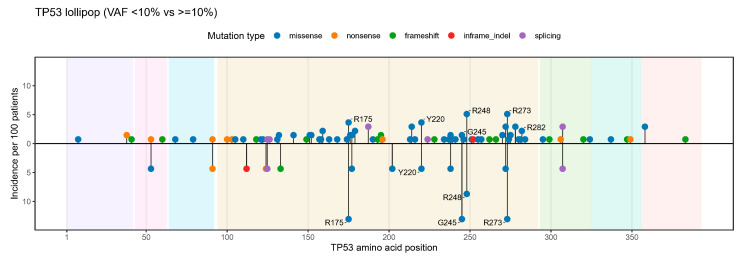
Distribution of dominant TP53 mutations stratified by VAF in TP53-mutated AML patients. Two-panel lollipop plots summarize one dominant mutation per patient (upper: VAF ≥ 10%; lower: VAF < 10%). Stem length encodes site-level frequency (% of patients within each group), and points are colored by mutation class (missense, nonsense, frameshift, splice, in-frame indel). Canonical hotspots (R175, Y220, G245, R248, R273, R282) are labeled. Shaded background regions indicate TP53 functional domains (TAD1, TAD2, PRD, DBD, Hinge, OD, and CTD).

Dominant TP53 mutations were mainly located in the DNA-binding domain (82.6% vs. 74.5%; *p* = 0.688), with missense variants being most frequent (73.9% vs. 71.9%; *p* = 1.000). Of note, classical TP53 hotspot mutations (R175, Y220, G245, R248, R273, R282) were significantly more frequent in the VAF < 10% group than in the VAF ≥ 10% group (52.2% vs. 20.7%; *p* = 0.003) (Figure 3).

### 3.4. Treatment and Outcomes

Among the 101 treated patients with available follow-up, 4 (3.96%) died within 30 days of therapy. Induction regimens were predominantly HMA ± VEN, and were well balanced between groups (76.9% vs. 76.2%; *p* = 1.000) (Table 2). Compared with the VAF ≥ 10% group, patients in the VAF < 10% group achieved a higher complete remission (CR) rate after the first course of induction (69.2% vs. 44.0%; *p* = 0.135), although the difference did not reach statistical significance. Overall CR rate, receiving allo-HSCT, and mortality were also non-significant (Table 2).

**Figure 4:**
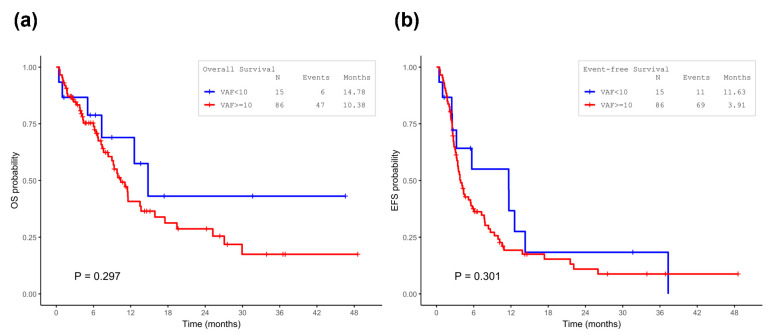
Kaplan–Meier survival analysis of TP53-mutated AML patients stratified by VAF. (**a**) Overall survival (OS) in AML patients with TP53 mutations, stratified by dominant TP53 VAF (<10% vs. ≥10%). Patients with VAF < 10% showed a trend toward improved outcomes compared with those with VAF ≥ 10%, although the differences did not reach statistical significance (OS, *p* = 0.297); (**b**) Fvent-free survival (EFS) in AML patients with TP53 mutations, stratified by dominant TP53 VAF (<10% vs. ≥10%). Patients with VAF < 10% showed a trend toward improved outcomes compared with those with VAF ≥ 10%, although the differences did not reach statistical significance (EFS, *p* = 0.301).

The median follow-up time was 14.6 months (95% CI, 8.4–20.7 months). In survival analyses, OS was numerically longer in the VAF < 10% group than in the VAF ≥ 10% group (*p* = 0.297; Figure 4a). A similar trend was observed in EFS (*p* = 0.301; Figure 4b). Among patients who achieved CR, the VAF < 10% group also had numerically superior RFS than the high-VAF group (*p* = 0.351; Figure S2). Overall, we did not observe a statistically significant difference in survival between the VAF < 10% and VAF ≥ 10% groups, but the small and imbalanced subgroup size limited statistical power.

### 3.5. Sensitivity Analysis Using a 20% TP53 VAF Cutoff

Because a 20% TP53 VAF threshold is frequently used in prior reports [6,13,22], we performed a sensitivity analysis using 20% to test the robustness of our findings. 160 patients with TP53 mutations were stratified by dominant TP53 VAF at diagnosis into VAF < 20% (N = 30, 18.8%) and VAF ≥ 20% (N = 130, 81.3%) groups, and baseline clinical and genetic characteristics are provided in Table S2.

Compared with the VAF ≥ 20% group, patients with VAF < 20% had lower WBC counts (median: 1.94 vs. 4.20; *p* = 0.002), lower blast percentage in bone marrow (median: 32.0% vs. 41.0%; *p* = 0.023) and peripheral blood (median: 2.0% vs. 23.0%; *p* < 0.001), higher proportion of CD34^+^CD38^−^ blast cells (35.7% vs. 15.0%; *p* = 0.016), a lower incidence of complex karyotype (37.9% vs. 79.1%; *p* < 0.001), monosomy 5/del(5q) (17.2% vs. 54.3%; *p* < 0.001) and 17p abnormality (13.8% vs. 34.6%; *p* = 0.028). In contrast, they had a higher number of co-mutations (median: 3 vs. 1, *p* = 0.002), more frequent ASXL1 (30.0% vs. 9.2%; *p* = 0.005) and SRSF2 (20.0% vs. 3.1%; *p* = 0.003) mutations, and a higher prevalence of TP53 hotspot mutations (50.0% vs. 19.5%; *p* = 0.002). No significant differences were observed for other variables, including age, sex, hemoglobin, platelet count, monosomy 7, TP53 mutation site (DBD vs. non-DBD), and TP53 mutation count.

Among the 101 patients with evaluable treatment and outcome data (VAF < 20%, N = 20; VAF ≥ 20%, N = 81), the use of HMA ± VEN for induction therapy was comparable between groups (83.3% vs. 74.7%; *p* = 0.550). However, the VAF < 20% group achieved a significantly higher CR rate after the first induction course (72.2% vs. 41.8%; *p* = 0.034). No statistically significant differences were found in CR rate, allo-HSCT rate and death rate (*p* = 0.179, 0.386, and 0.436, respectively).

Overall, the results using the 20% cutoff were directionally consistent with those obtained using the 10% cutoff.

## 4. Discussion

The biological and prognostic significance of TP53 VAF in AML has not been fully elucidated. While the ICC currently adopts an empirical 10% threshold and other studies have explored alternative cutoffs such as 20–23% with mixed results [6,10,11,12], it remains unclear whether low-VAF TP53 mutations define a distinct subgroup. Based on our real-world, single-center cohort of TP53-mutated AML, and stratifying patients using 10% dominant TP53 VAF cutoff, we observed that the VAF < 10% group exhibited several distinctive biological features: a higher proportion of LSCs (CD34^+^CD38^−^ blast immunophenotype), a lower incidence of adverse cytogenetic abnormalities, and a higher frequency of adverse molecular alterations (notably ASXL1 and SRSF2 mutations, and EVI1 overexpression). Concomitantly, TP53 mutations were more frequently located at hotspot sites. Regarding prognosis, both groups showed dismal prognosis and no significant differences were observed across OS, EFS and RFS, although the limited sample size and imbalanced group allocation may have con-strained statistical power. We further repeated the comparison using a 20% cutoff as a sensitivity analysis, and the results were directionally concordant with those using 10%, supporting the robustness of our conclusions.

Prochazka and colleagues grouped TP53-mutated AML by VAF < 20%, 20–40%, and ≥40%, and found that the <20% group had a significantly lower incidence of complex karyotype than the other two groups [13]. Our findings are consistent, demonstrating that the proportion of complex karyotype was lower in the VAF < 10% group. In addition, we observed that other adverse cytogenetic abnormalities. Monosomy 5/del(5q) and monosomy 7 were significantly less frequent in the VAF < 10% group. By contrast, abnormalities involving 17p tended to occur less often, with *p* values approaching but not reaching statistical significance. Biologically, TP53 is a tumor suppressor that plays a critical role in maintaining genome stability. Previous studies have consistently demonstrated that TP53-mutated myeloid neoplasms are frequently associated with widespread cytogenetic abnormalities, particularly complex karyotype and monosomy or deletions involving chromosomes 5 and 7, which are generally thought to arise from chromosome instability caused by loss of TP53 function [22]. In this context, a higher TP53 VAF often suggests that the TP53 mutation represents an earlier and more dominant clonal event during leukemogenesis, thereby facilitating the accumulation of complex chromosomal abnormalities.

Despite this relatively lower burden of adverse cytogenetic alterations, low-VAF patients displayed more unfavorable molecular characteristics, including a higher number of co-mutated genes, increased frequencies of mutations in ASXL1 and SRSF2 [21,23,24], and a higher prevalence of EVI1 overexpression [25,26]. In our cohort, the VAF < 10% group showed a numerically higher proportion of secondary AML (43.5% vs. 27.0%). Secondary AML is frequently enriched for clonal hematopoiesis/MDS-related mutations, including *ASXL1* and spliceosome genes (e.g., *SRSF2*) [27], consistent with our findings. Clinically, secondary AML/AML with myelodysplasia-related features is associated with inferior treatment response and adverse outcomes and is recognized as a distinct entity in contemporary classification systems [15,28,29]. Therefore, the higher proportion of secondary AML in the low-VAF group may partly contribute to our OS results, and survival should be interpreted in the context of AML ontogeny rather than TP53 VAF alone. Collectively, these findings suggest that low-VAF TP53 AML may constitute a subgroup characterized by adverse molecular features. Given the limited sample size, future studies with larger cohorts of low-VAF TP53 patients are required to further delineate this subtype and to better define the optimal VAF threshold.

Chemoresistance is a critical determinant of poor prognosis in AML, with subclonal expansion considered an important mechanism [30]. Yan et al. demonstrated in two AML cell lines harboring low-frequency TP53 mutations (OCI-AML2: Y220C; MV4-11: R248W) that these low-VAF mutations were closely associated with the acquisition of chemoresistance [31]. During the development of resistance, cell populations with a LSC phenotype expanded significantly. Previous studies have shown that hotspot mutations (R175, Y220, G245, R248, R273, R282), in addition to causing loss of p53 function, can exert dominant-negative effects, and through gain-of-function (GOF) properties, further suppress p53 function and enhance in vivo fitness [32,33]. Furthermore, the GOF activity of p53 can upregulate the expression of multiple cancer stem cell–related genes, activate survival and resistance-related pathways, and markedly promote invasiveness and chemoresistance [34,35,36]. Consistent with this, we observed a higher frequency of hotspot mutations and a higher proportion of CD34^+^CD38^−^ LSCs in patients with VAF < 10%. Residual LSCs are thought to seed MRD and drive relapse. Although LSCs can reside within CD34^+^CD38^+^, CD34^+^CD38^−^, and CD34^−^ subsets, the CD34^+^CD38^−^ compartment is the most therapy-resistant and least immunogenic, serving as a strong predictor of relapse [37,38]. These may partly explain our observation that low-VAF patients had higher CR rates after induction therapy but still resulted in comparable overall CR and poor long-term outcomes (OS, EFS and RFS), indicating that low VAF did not confer a clear survival advantage. These findings suggest that low VAF does not necessarily equate to reduced biological aggressiveness. Instead, such subclones may drive relapse by enhancing stemness and GOF effects. Nevertheless, these mechanisms require validation in larger cohorts and functional studies.

According to the ICC criteria, TP53 mutations are considered only when VAF > 10%. Accordingly, the 23 patients with TP53 VAF < 10% were not considered TP53-mutated. Risk stratification was then performed based on their other cytogenetic and molecular features in accordance with the 2022 ELN guidelines [21]. The results showed that, even without considering TP53 mutations, 78.3% (18/23) of patients were still categorized in the adverse-risk group due to other factors (Figure S3; Table S3). Of the remaining five patients, two were classified as favorable-risk because of RUNX1::RUNX1T1 positivity and an NPM1 mutation, respectively. The former relapsed within one year of initial therapy. The latter, an older adult, presented with hyperleukocytosis (WBC > 100 × 10^9^/L) [39], excess bone marrow blasts [39], and a co-occurring DNMT3A mutation [40], all linked to adverse prognosis in NPM1-mutated AML. However, follow-up to date is limited and continued observation is warranted. Taken together, these data indicate that while TP53-mutated AML patients with low-VAF may have slightly better outcomes than those with VAF ≥ 10%, their overall prognosis remains poor due to distinct adverse biological features.

This study has limitations. First, its retrospective single-center design is susceptible to selection bias. Second, given the overall low incidence of TP53 mutations, our sample size was limited. Third, our focus on the 10% cutoff resulted in markedly imbalanced group sizes, which constrained statistical efficiency. The small number of events in the VAF < 10% group and the imbalanced allocation limited detectability, such that moderate survival effects may not reach statistical significance. To mitigate bias, we preferentially used Fisher’s exact tests for comparisons of rates and conducted a sensitivity analysis using a 20% cutoff, which yielded consistent results. Future work in larger cohorts should incorporate multivariable models while adjusting for age, complex karyotype, EVI1, induction regimen and HSCT, to more precisely delineate the statistical and clinical relevance of VAF thresholds.

In summary, TP53-mutated AML patients with VAF < 10% may represent a distinct subtype with unique clinical and genetic features. Although they exhibit lower incidences of adverse cytogenetic alterations, higher frequencies of adverse molecular characteristics and a higher proportion of CD34^+^CD38^−^ blast immunophenotype confer an overall poor prognosis. The sensitivity analysis using a 20% cutoff yielded consistent results. Collectively, these findings suggest that low-VAF TP53 mutations should not be overlooked, and that the biological heterogeneity and clinical risk of this subgroup underscore the need for validation in larger cohorts and prospective studies.

## Figures and Tables

**Table 1:** Baseline features of TP53-mutated AML by VAF (<10% vs. ≥10%).

	Total (N = 160)	VAF < 10% (N = 23)	VAF ≥ 10% (N = 137)	*p*-Value
Sex (male), n (%)	91 (56.9%)	17 (73.9%)	74 (54.0%)	0.110
Age, median (range)	61 (18–88)	63 (39–88)	61 (18–85)	0.174
AML type				0.236
de novo	100 (62.5%)	11 (47.8%)	89 (65.0%)	
secondary	47 (29.4%)	10 (43.5%)	37 (27.0%)	
therapy-related	13 (8.1%)	2 (8.7%)	11 (8.0%)	
Blood counts, median (range)				
WBC count, ×10^9^/L	3.63 (0.64–124.01)	2.00 (0.88–121.18)	4.00 (0.64–124.01)	0.045
Hb, g/L	75 (40–159)	88 (48–159)	74 (40–134)	0.067
PLT, ×10^9^/L	43.5 (1–973)	62.0 (4–973)	39.0 (1–506)	0.115
Blast% in BM	39.0 (8–97)	36.0 (12–78)	39.8 (8–97)	0.223
Blast% in PB	19.0 (0–89)	2.0 (0–79)	22.5 (0–89)	<0.001
Immunophenotypic markers				
CD34^+^	139 (88.5%)	20 (90.9%)	119 (88.1%)	1.000
CD38^+^	124 (80.0%)	12 (57.1%)	112 (83.6%)	0.015
CD34^+^CD38^−^	29 (18.7%)	8 (38.1%)	21 (15.7%)	0.030
CD117^+^	152 (96.2%)	23 (100.0%)	129 (95.6%)	0.594
CD33^+^	145 (94.8%)	22 (100.0%)	123 (93.9%)	0.603
CD13^+^	144 (94.7%)	18 (90.0%)	126 (95.5%)	0.284
HLA-DR^+^	128 (88.3%)	16 (94.1%)	112 (87.5%)	0.694
CD7^+^	44 (30.8%)	6 (37.5%)	38 (29.9%)	0.571
CD123^+^	77 (53.5%)	10 (62.5%)	67 (52.3%)	0.597
Complex karyotype, n (%)	113 (71.5%)	7 (31.8%)	106 (77.9%)	<0.001
Monosomy 7	38 (24.1%)	1 (4.5%)	37 (27.2%)	0.028
Monosomy 5/del(5q)	75 (47.5%)	1 (4.5%)	74 (54.4%)	<0.001
17p abnormality, n (%) *	48 (30.8%)	3 (13.6%)	45 (33.6%)	0.080
WT1 (≥0.6%), n (%)	138 (86.8%)	21 (91.3%)	117 (86.0%)	0.741
EVI1 (≥8.0%), n (%)	38 (24.2%)	10 (45.5%)	28 (20.7%)	0.028
Co-mutation count (I-II), median (range)	2 (0–9)	3 (0–8)	1 (0–9)	0.001
NPM1	12 (7.5%)	2 (8.7%)	10 (7.3%)	0.684
FLT3-ITD	5 (3.1%)	2 (8.7%)	3 (2.2%)	0.151
KIT	2 (1.3%)	0 (0.0%)	2 (1.5%)	1.000
CEBPA bZIP	2 (1.3%)	0 (0.0%)	2 (1.5%)	1.000
ASXL1	21 (13.1%)	7 (30.4%)	14 (10.2%)	0.015
BCOR	4 (2.5%)	1 (4.3%)	3 (2.2%)	0.466
EZH2	6 (3.8%)	1 (4.3%)	5 (3.6%)	1.000
RUNX1	9 (5.6%)	3 (13.0%)	6 (4.4%)	0.122
SF3B1	4 (2.5%)	1 (4.3%)	3 (2.2%)	0.466
SRSF2	10 (6.3%)	6 (26.1%)	4 (2.9%)	0.001
STAG2	6 (3.8%)	2 (8.7%)	4 (2.9%)	0.207
U2AF1	8 (5.0%)	2 (8.7%)	6 (4.4%)	0.323
ZRSR2	1 (0.6%)	1 (4.3%)	0 (0.0%)	0.144
DNMT3A	19 (11.9%)	1 (4.3%)	18 (13.1%)	0.314
TET2	15 (9.4%)	2 (8.7%)	13 (9.5%)	1.000
KRAS	11 (6.9%)	2 (8.7%)	10 (7.3%)	0.684
NRAS	18 (11.3%)	1 (4.3%)	17 (12.4%)	0.474
IDH1	12 (7.5%)	1 (4.3%)	11 (8.0%)	1.000
IDH2	9 (5.6%)	2 (8.7%)	7 (5.1%)	0.618
TP53 mutation count				
≥2, n (%)	30 (18.8%)	2 (8.7%)	28 (20.4%)	0.253
median (range)	1 (1–3)	1 (1–2)	1 (1–3)	0.177
TP53 mutation site				
DBD	121 (75.6%)	19 (82.6%)	102 (74.5%)	0.688
TP53 mutation type				
Missense	114 (72.2%)	17 (73.9%)	97 (71.9%)	1.000
Hotspot, n (%)	40 (25.3%)	12 (52.2%)	28 (20.7%)	0.003

* 17p abnormality includes monosomy 17, del(17p), cn-LOH(17p).

**Table 2:** Treatment and outcomes of TP53-mutated AML by VAF (<10% vs. ≥10%).

Treatment and Outcome	Total (N = 101)	VAF < 10% (N = 15)	VAF ≥ 10% (N = 86)	*p*-Value
Induction therapy (n = 97)				1.000
HMA ± VEN	74 (76.3%)	10 (76.9%)	64 (76.2%)	
Others	23 (23.7%)	3 (23.1%)	20 (23.8%)	
CR after C1, n (%) (n = 97) *	46 (47.4%)	9 (69.2%)	37 (44.0%)	0.135
CR, n (%)	60 (61.9%)	10 (76.9%)	50 (59.5%)	0.359
Allo-HSCT, n (%)	23 (22.8%)	5 (33.3%)	18 (20.9%)	0.322
Death, n (%)	34 (33.7%)	3 (20.0%)	31 (36.0%)	0.374

* CR after C1 denotes patients achieving CR, CRh, or CRi after the first induction cycle.

## Data Availability

Data are not available owing to ethical restrictions.

## Supplementary Materials

The following supporting information can be downloaded at: https://www.mdpi.com/article/10.3390/biomedicines14010180/s1, Figure S1: Comparison of clinical, cytogenetic, and molecular features between AML patients with dominant TP53 VAF < 10% and ≥10%; Figure S2: Kaplan–Meier survival analysis of TP53-mutated AML patients stratified by VAF (RFS); Figure S3: Landscape of cytogenetic and molecular features in VAF < 10% TP53-mutated AML (N = 23); Figure S4: Kaplan–Meier survival analysis of TP53-mutated AML patients receiving allo-HSCT stratified by TP53 VAF (OS); Table S1: Genes included in the 139-gene myeloid targeted NGS panel; Table S2: Baseline features and outcomes of TP53-mutated AML by VAF (<20% vs. ≥20%); Table S3: Cytogenetic and molecular profiles and ELN 2022 risk classification excluding TP53 status in VAF < 10% TP53-mutated AML (N = 23).

## Abbreviations

The following abbreviations are used in this manuscript:

AML	Acute myeloid leukemia
VAF	Variant allele frequency
CH	Clonal hematopoiesis
NGS	Next-generation sequencing
