# Supplementary material for: Low-VAF TP53-Mutated AML Displays Distinct Biological Features in a Single-Center Cohort
Source: Biomedicines. 2026 Jan 14;14(1):180. doi: 10.3390/biomedicines14010180 (PMC12839384; doi:10.3390/biomedicines14010180)
Supplement: Supplementary file 1 [file biomedicines-14-00180-s001.zip › biomedicines-4047568-supplementary.pdf]

Title

Low-VAF TP53-Mutated AML Displays Distinct Biological Features in a Single-Center Cohort

Xiaoxuan Lu [1], Xiaohang Ma [1], Kainan Zhang [1], Shun Zhang [1], Fangfang Wei [1], Hao Jiang [1], Qian Jiang [1], Yingjun Chang [1], Xiaojun Huang [1,2,3,4] and Xiaosu Zhao [1,3,4,*]

[1] Beijing Key Laboratory of Cell and Gene Therapy for Hematologic Malignancies, Peking University People's Hospital, Peking University Institute of Hematology, National Clinical Research Center for Hematologic Disease, Peking University, Beijing 100044, China; luxx1005@163.com (X.L.)

[2] Peking-Tsinghua Center for Life Sciences, Academy for Advanced Interdisciplinary Studies, Peking University, Beijing 100871, China

[3] Research Unit of Key Technique for Diagnosis and Treatments of Hematologic Malignancies, Chinese Academy of Medical Sciences, 2019RU029, Beijing 100730, China

[4] Collaborative Innovation Center of Hematology, Peking University, Beijing 100044, China

* Correspondence: zhao.xiaosu@outlook.com

Supplementary File

Supplementary materials

Supplementary Figure S1. Comparison of clinical, cytogenetic, and molecular features between AML patients with dominant *TP53* VAF <10% and ≥10%.

Supplementary Figure S2. Kaplan–Meier survival analysis of *TP53*-mutated AML patients stratified by VAF (RFS).

Supplementary Figure S3. Landscape of cytogenetic and molecular features in VAF <10% *TP53*-mutated AML (n=23).

Supplementary Figure S4. Kaplan–Meier survival analysis of *TP53*-mutated AML patients receiving allo-HSCT stratified by *TP53* VAF (OS).

Supplementary Table S1. Genes included in the 139-gene myeloid targeted NGS panel.

Supplementary Table S2. Baseline features and outcomes of *TP53*-mutated AML by VAF (<20% vs ≥20%).

Supplementary Table S3. Cytogenetic and molecular profiles and ELN 2022 risk classification excluding *TP53* status in VAF <10% *TP53*-mutated AML (n=23).

Supplementary materials

TP53 mutation detection by high-depth targeted next-generation sequencing

High-depth targeted next-generation sequencing (NGS) was performed on diagnostic samples obtained at the time of initial AML diagnosis. Genomic DNA was extracted from bone marrow or peripheral blood samples using standard protocols. Targeted sequencing was conducted using a customized myeloid malignancy panel covering 139 genes recurrently mutated in myeloid neoplasms (gene list provided in Supplementary Table S1).

Library preparation was performed using a hybrid capture–based enrichment strategy. The captured libraries were sequenced on an Illumina NovaSeq platform to generate 150 bp paired-end reads. High sequencing depth was achieved across targeted regions to enable reliable detection of low-frequency variants. Single-nucleotide variants (SNVs) and short insertions/deletions (indels) were identified using routinely applied bioinformatics pipelines, and variant annotation was performed using established public databases.

To ensure accuracy of variant calling, only variants meeting the following quality criteria were retained for analysis: an average effective sequencing depth of ≥2000× in target regions, sufficient read and base quality scores, and a variant allele frequency (VAF) ≥1%. Variants with a VAF <1% were excluded from downstream analyses.

TP53 mutations included missense, nonsense, frameshift, and canonical splice-site variants. TP53 allelic status was comprehensively assessed by integrating targeted NGS results with conventional cytogenetic analysis, fluorescence in situ hybridization (FISH), and copy number variation (CNV) assessment, as described in the main text.

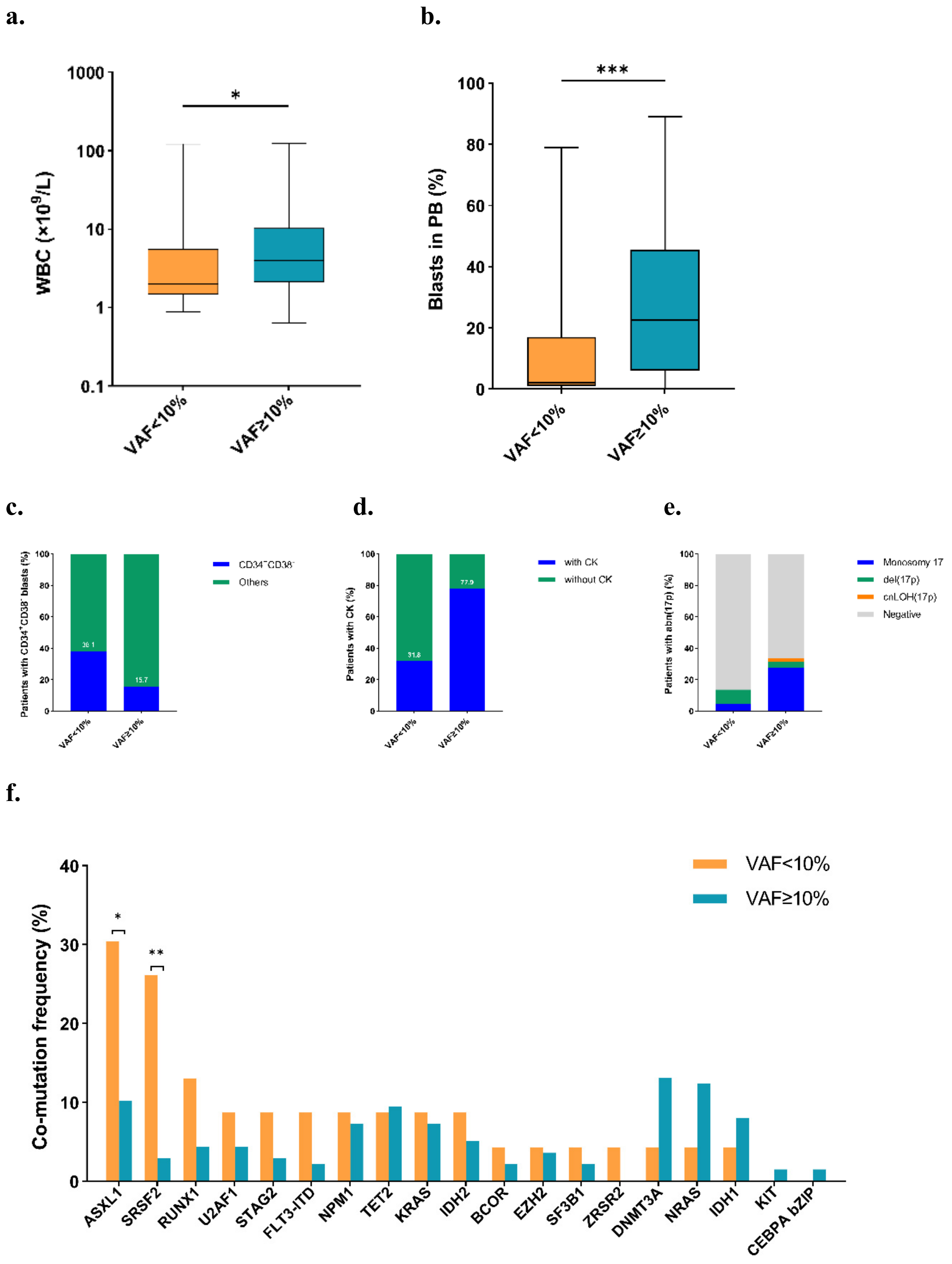

Supplementary Figure S1. Comparison of clinical, cytogenetic, and molecular features between AML patients with dominant *TP53* VAF <10% and ≥10%.

(a) White blood cell count (y-axis on a log10 scale).

(b) Blast percentage in peripheral blood.

(c) AML blasts with the CD34/CD38 immunophenotype ($CD34^{+}CD38^{-}$).

(d) Complex karyotype.

(e) 17p abnormality.

(f) Co-mutation frequency for the indicated genes.

* Continuous variables are displayed as boxplots or grouped column chart; categorical variables are shown as within-group proportions (Percentages stacked bars chart).

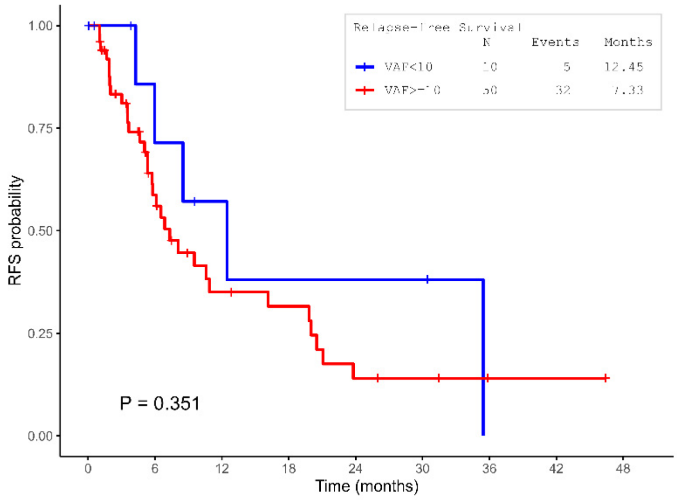

Supplementary Figure S2. Kaplan–Meier survival analysis of *TP53*-mutated AML patients stratified by VAF.

Relapse-free survival (RFS) in AML patients with *TP53* mutations, stratified by dominant *TP53* VAF (<10% vs ≥10%).

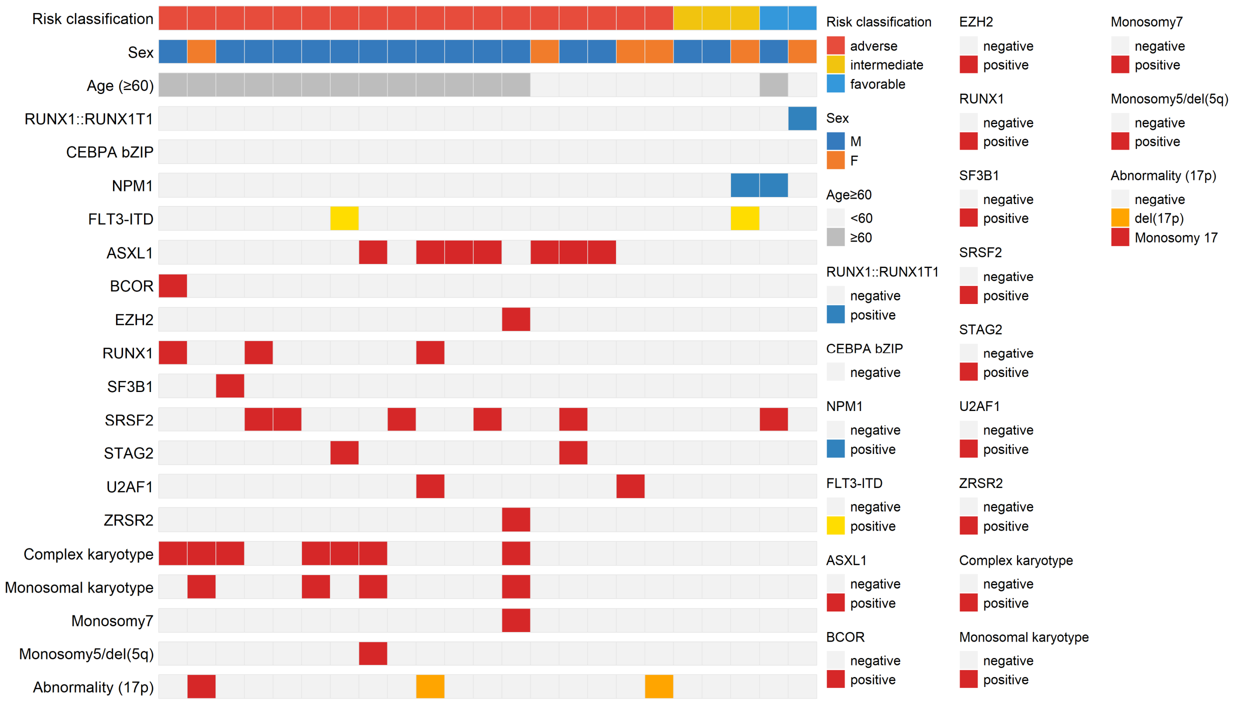

Supplementary Figure S3. Landscape of cytogenetic and molecular features in VAF <10% *TP53*-mutated AML (n=23).

Each column represents one patient. Rows display ELN2022 risk classification, sex, age, gene fusions/mutations (*RUNX1::RUNX1T1, CEBPA bZIP* in frame, *NPM1, FLT3-ITD, ASXL1, BCOR, EZH2, RUNX1, SF3B1, SRSF2, STAG2, U2AF1, ZRSR2*) and cytogenetic abnormalities [complex karyotype, monosomal karyotype, monosomy 7, monosomy 5/del(5q), and 17p abnormalities].

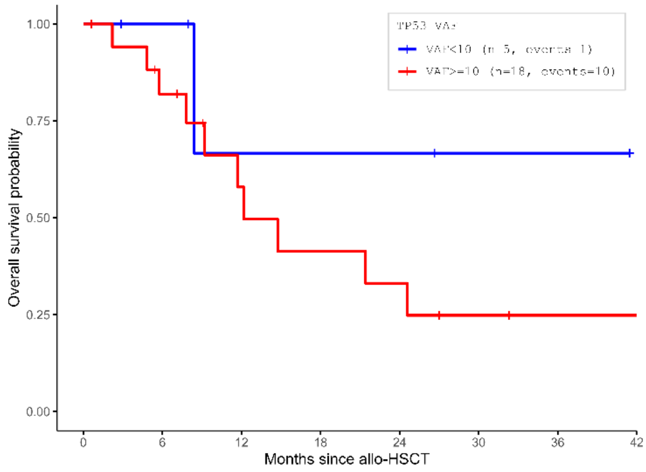

Supplementary Figure S4. Kaplan–Meier survival analysis of TP53-mutated AML patients receiving allo-HSCT stratified by TP53 VAF (OS).

Overall survival (OS) after allogeneic hematopoietic stem cell transplantation (allo-HSCT) in AML patients with TP53 mutations, stratified by dominant TP53 variant allele frequency (VAF <10% vs ≥10%).

Supplementary Table S1. Genes included in the 139-gene myeloid targeted NGS panel.

ABCB1	*BRAF*	*CTCF*	*FLT3*	*JAK1*	*NRAS*	*SETBP1*	*TCF3*
ABL1	*BRCA1*	*CUX1*	*G6PC3*	*JAK2*	*NT5C2*	*SETD2*	*TERC*
AKT3	*BRCA2*	*DDX41*	*GATA1*	*JAK3*	*NTRK1*	*SETDB1*	*TERT*
ANKRD26	*BRIP1*	*DIS3*	*GATA2*	*KDM6A*	*PAX5*	*SF1*	*TET1*
ARID1A	*CALR*	*DKC1*	*GATA3*	*KIT*	*PDGFRA*	*SF3A1*	*TET2*
ARID1B	*CBL*	*DNMT3A*	*GFI1*	*KMT2A*	*PDGFRB*	*SF3B1*	*TP53*
ARID2	*CBLB*	*EED*	*GNAS*	*KMT2B*	*PHF6*	*SH2B3*	*TPMT*
ASXL1	*CBLC*	*EGFR*	*GNB1*	*KMT2C*	*PIGA*	*SMC1A*	*U2AF1*
ASXL2	*CCND3*	*EGLN1*	*GSKIP*	*KMT2D*	*PML*	*SMC3*	*U2AF2*
ATG2B	*CDKN1A*	*ELANE*	*HAX1*	*KRAS*	*PPM1D*	*SOCS1*	*VHL*
ATM	*CDKN2A*	*EP300*	*HRAS*	*LMO2*	*PRPF8*	*SRP72*	*WAS*
ATRX	*CDKN2B*	*EPOR*	*ID3*	*MPL*	*PTEN*	*SRSF2*	*WT1*
BCL2	*CEBPA*	*ERG*	*IDH1*	*MYC*	*PTPN11*	*STAG2*	*ZRSR2*
BCL6	*CHEK2*	*ETNK1*	*IDH2*	*NBN*	*RAD21*	*STAT3*	
BCOR	*CREBBP*	*ETV6*	*IKZF1*	*NF1*	*RARA*	*STAT5A*	
BCORL1	*CRLF2*	*EZH2*	*IKZF2*	*NOTCH1*	*RB1*	*STAT5B*	
BLM	*CSF1R*	*FBXW7*	*IKZF3*	*NOTCH2*	*RUNX1*	*SUZ12*	
BPGM	*CSF3R*	*FGFR3*	*IL7R*	*NPM1*	*SBDS*	*TAL1*	

Supplementary Table S2. Baseline features and outcomes of *TP53*-mutated AML by VAF (<20% vs ≥20%).

	Total (N=160)	VAF<20% (n=30)	VAF≥20% (n=130)	P-value
**Sex (male), n (%)**	91 (56.9%)	22 (73.3%)	69 (53.1%)	0.065
**Age, median (range)**	61 (18-88)	62.5 (39-88)	61 (18-85)	0.141
**AML type**				0.076
de novo	100 (62.5%)	14 (46.7%)	86 (66.2%)	
secondary	47 (29.4%)	14 (46.7%)	33 (25.4%)	
therapy-related	13 (8.1%)	2 (6.7%)	11 (8.5%)	
**Blood counts, median (range)**				
WBC count, $\times10^9$/L	3.625 (0.64-124.01)	1.94 (0.88-121.18)	4.20 (0.64-124.01)	**0.002**
Hb, g/L	75 (40-159)	82 (48-159)	74.5 (40-134)	0.298
PLT, $\times10^9$/L	43.5 (1-973)	58.5 (4-973)	38.5 (1-506)	0.082
**Blast% in BM, median (range)**	39 (8-97)	32 (11-78)	41 (8-97)	**0.023**
**Blast% in PB, median (range)**	19 (0-89)	2 (0-79)	23 (0-89)	**<0.001**
**Immunophenotypic markers**				
$CD34^+$	139 (88.5%)	27 (93.1%)	112 (87.5%)	0.530
$CD38^+$	124 (80.0%)	17 (60.7%)	107 (84.3%)	**0.008**
$CD34^+CD38^-$	29 (18.7%)	10 (35.7%)	19 (15.0%)	**0.016**
$CD117^+$	152 (96.2%)	30 (100.0%)	122 (95.3%)	0.596
$CD33^+$	145 (94.8%)	28 (96.6%)	117 (94.4%)	1.000
$CD13^+$	144 (94.7%)	25 (92.6%)	119 (95.2%)	0.633
HLA-DR^+	128 (88.3%)	23 (95.8%)	105 (86.8%)	0.307
$CD7^+$	44 (30.8%)	6 (27.3%)	38 (31.4%)	0.805
$CD123^+$	77 (53.5%)	14 (60.9%)	63 (52.1%)	0.499
**Complex karyotype, n (%)**	113 (71.5%)	11 (37.9%)	102 (79.1%)	**<0.001**
**Monosomy 7, n (%)**	38 (24.1%)	3 (10.3%)	35 (27.1%)	0.059
**Monosomy 5/ del(5q), n (%)**	75 (47.5%)	5 (17.2%)	70 (54.3%)	**<0.001**
**17p abnormality, n (%) ***	48 (30.8%)	4 (13.8%)	44 (34.6%)	**0.028**
***WT1* (≥0.6%), n (%)**	138 (86.8%)	26 (86.7%)	112 (86.8%)	1.000
***EVI1* (≥8.0%), n (%)**	38 (24.2%)	12 (41.4%)	26 (20.3%)	**0.029**
**Co-mutation count (I-II), median (range)**	2 (0-9)	3 (0-8)	1 (0-9)	**0.002**
NPM1	12 (7.5%)	2 (6.7%)	10 (7.7%)	1.000
FLT3-ITD	5 (3.1%)	2 (6.7%)	3 (2.3%)	0.236
KIT	2 (1.3%)	0 (0.0%)	2 (1.5%)	1.000
CEBPA bZIP	2 (1.3%)	0 (0.0%)	2 (1.5%)	1.000
ASXL1	21 (13.1%)	9 (30.0%)	12 (9.2%)	**0.005**
BCOR	4 (2.5%)	2 (6.7%)	2 (1.5%)	0.160
EZH2	6 (3.8%)	1 (3.3%)	5 (3.8%)	1.000
RUNX1	9 (5.6%)	3 (10.0%)	6 (4.6%)	0.371
SF3B1	4 (2.5%)	2 (6.7%)	2 (1.5%)	0.160

SRSF2	10 (6.3%)	6 (20.0%)	4 (3.1%)	**0.003**
STAG2	6 (3.8%)	3 (10.0%)	3 (2.3%)	0.080
U2AF1	8 (5.0%)	3 (10.0%)	5 (3.8%)	0.172
ZRSR2	1 (0.6%)	1 (3.3%)	0 (0.0%)	0.187
DNMT3A	19 (11.9%)	4 (13.3%)	15 (11.5%)	0.758
TET2	15 (9.4%)	2 (6.7%)	13 (10.0%)	0.739
KRAS	11 (6.9%)	2 (6.7%)	10 (7.7%)	1.000
NRAS	18 (11.3%)	2 (6.7%)	16 (12.3%)	0.529
IDH1	12 (7.5%)	3 (10.0%)	9 (6.9%)	0.699
IDH2	9 (5.6%)	2 (6.7%)	7 (5.4%)	0.676
***TP53* mutation count**				
≥2, n (%)	30 (18.8%)	4 (13.3%)	26 (20.0%)	0.604
median (range)	1 (1-3)	1 (1-2)	1 (1-3)	0.380
***TP53* mutation site**				
DBD	121 (75.6%)	25 (83.3%)	96 (73.8%)	0.418
***TP53* mutation type**				
Missense	114 (72.2%)	22 (73.3%)	92 (71.9%)	1.000
**Hotspot, n (%)**	40 (25.3%)	15 (50.0%)	25 (19.5%)	**0.002**

* Data are n (%) unless otherwise indicated; continuous variables are reported as median (range). P values were calculated using Fisher's exact test or the Wilcoxon rank-sum test, as appropriate.

**Treatment and outcome**	**Total (N=101)**	**VAF<20% (n=20)**	**VAF≥20% (n=81)**	**P-value**
**Induction therapy (n=97)**				0.550
HMA±VEN	74 (76.3%)	15 (83.3%)	59 (74.7%)	
Others	23 (23.7%)	3 (16.7%)	20 (25.3%)	
**CR after C1, n (%) (n=97)**	46 (47.4%)	13 (72.2%)	33 (41.8%)	**0.034**
**CR, n (%)**	60 (61.9%)	14 (77.8%)	46 (58.2%)	0.179
**Allo-HSCT, n (%)**	23 (22.8%)	6 (30.0%)	17 (21.0%)	0.386
**Death, n (%)**	34 (33.7%)	5 (25.0%)	29 (35.8%)	0.436

Supplementary Table S3. Cytogenetic and molecular profiles and ELN 2022 risk classification excluding *TP53* status in VAF <10% *TP53*-mutated AML (n=23).

Patient id	sex	age	*TP53* Mutation count	*TP53* VAF	Risk classification	Monosomy 7	Monosomy 5/del(5q)	Monosomal karyotype	Complex karyotype	abn(17p)	EVI 1	RUNX1::RUNX1T1	NGS_Tier I/II
P116	F	53	1	1.20%	Adverse	0	0	0	Non-complex	No	862.20	Negative	*U2AF1*(p.R156H)8.7%; *KRAS*(p.A59T)3.2%; *KDM6A*(p.R658*)1.1%
P069	M	74	1	1.34%	Adverse	0	0	0	Non-complex	No	Normal	Negative	*SRSF2*(p.P95R)19.2%; *PHF6*(p.Q114*)30.7%; *RUNX1*(p.F330fs)11.4%; *RUNX1*(p.S141L)17.5%
P299	F	59	1	1.37%	Adverse	0	0	0	Normal	No	42.20	Negative	*ASXL1*(p.G646fs)34.7%; *CALR*-type I(p.L367fs) 23.2%; *NRAS*(p.G12V) 1.22%; *PRPF8*(p.D1598N) 41.4%
P073	M	66	1	1.40%	Adverse	0	0	0	Normal	No	106.80	Negative	*SRSF2*(p.P95T)11%; *DDX41*(c.1549+1G>T)47.8%; *DDX41*(p.R525H)8.6%; *CUX1*(p.P522fs)1.7%;

P295	M	69	1	1.50%	**Favorable**	0	0	0	Normal	No	Nor mal	Negative	*DNMT3A*(p.R882H)%; *IDH2*(p.R140Q)%; *NF1*(p.M102fs)%; *NPM1*-A(p.W288fs)%; *SRSF2*(p.P95H)%; *TPMT*(p.Y240C)%; *ARID2*(p.E22*)%
P113	M	74	1	1.90%	Adverse	0	0	0	Normal	No	161. 60	Negative	*DDX41*(p.R525H)14.3%; *DDX41*(p.Q63*)48.5%; *SRSF2*(p.P95L)15.7%; *CUX1*(p.E266fs)2.1%
P074	M	63	1	2.10%	Adverse	0	0	0	Normal	No	75.9 0	Negative	*ASXL1*(p.E797*)44.6%; *CALR*(p.K385fs)81.7%; *PHF6*(p.R342*)89.7%; *BRAF*(p.G469A)2.6%; *SETBP1*(p.I871T)17.5%
P071	M	62	1	2.10%	Adverse	0	0	0	Normal	No	Nor mal	Negative	*ASXL1*(p.D864*)2.5%; *CEBPA*(p.E290*)37%; *CEBPA*(p.L78fs)33.5%; *SRSF2*(p.P95H)5.3%; *TET2*(p.Q341*)3%; *TET2*(p.R1516*)47.8%; *TET2*(p.S1593*)3.9%; *CSF3R*(p.T640N)1.8%; *RAD21*(p.T312fs)30.3%; *TET2*(p.P1194R)37.4%

P296	M	58	1	2.20%	Adverse	0	0	0	Non-complex	No	Normal	Negative	*ASXL1*(p.G869fs) 37.8%; *IDH2*(p.R140Q) 38.9%; *KRAS*(p.A146T) 6%; *SRSF2*(p.P95R) 38.5%; *STAG2*(c.1304+1_1304+5del) 28.5%; *CBL*(p.R149*) 4.7%; *CBL*(p.C396S) 2.7%; *CBL*(p.R420Q) 1.6%; *CEBPA*(p.L324P) 5%; *MED12*(p.Q1552*) 21%
P084	M	69	2	3.00%	Adverse	0	0	0	Complex	No	Normal	Negative	*FLT3-ITD* low; *STAG2*(c.816_819+7delinsC)2.4%; *TPMT*(p.Y240C)46%
P061	M	65	1	3.40%	Adverse	0	0	0	Non-complex	del(17p)	Normal	Negative	*ASXL1*(p.G646fs)35.2%; *U2AF1*(p.S34Y)36.7%; *EP300*(p.R1680fs)9.6%; *RUNX1*(p.R107C)1.6%
P298	M	88	1	4.50%	Adverse	0	0	0	Complex	No	NA	Negative	*BCOR*(p.K1687*)9%; *IDH1*(p.R132C) 4.1%; *RUNX1*(p.P113S) 8.6%; *SOCS1*(p.A3V) 46.2%
P081	M	73	1	5.00%	Adverse	0	0	1	Complex	No	14.50	Negative	None

P300	M	62	1	5.30%	Adverse	1	0	1	Complex	No	Nor mal	Negative	*ETV6*(p.L179fs)6.6%; *ETV6*(p.Q198*) 27.7%; *ZRSR2*(c.827+1G>A) 68.1%; *EZH2*(p.V626M) 37.50%
P107	M	76	1	6.10%	Adverse	0	0	0	Complex	No	113. 30	Negative	*SF3B1*(p.K700E)5.5%; *CBL*(p.Y371H)5.7%
P070	M	54	1	7.20%	Intermediate	0	0	0	Normal	No	62.3 0	Negative	*DDX41*(p.R525H) 3.90%; *DDX41*(p.S217fs) 45.20%。
P304	F	47	1	7.40%	Intermediate	0	0	0	Normal	No	Nor mal	Negative	*NPM1*-A(p.W288fs) 35.5%; *WT1*(p.R385fs)34.9%; *FLT3-ITD* (p.F612_G613 insDPREYEYDLKWEFPR ENLEF) 59.55%; *SETD2*(p.S717*) 8.4%
P089	M	57	1	7.50%	Adverse	NA	NA	NA	Unknown	NA	55.3 0	Negative	*ASXL1*(p.E635fs)19.4%; *TET2*(p.Q626fs)25.4%; *DDX41*(p.I310fs)40.1%; *DDX41*(p.R525H)13.2%; *PPM1D*(p.E424*)1.9%; *PHF6*(c.585+2T>C)9%; *CUX1*(p.Q351*)15.2%
P080	F	43	1	8.10%	Adverse	0	0	0	Normal	del(17p)	44.7 0	Negative	None
P108	F	79	1	8.10%	Adverse	0	0	1	Complex	-17	Nor	Negative	*STAT3*(p.D661V)1.30%

											mal		
P072	F	39	1	8.20%	**Favorable**	0	0	0	Non-complex	No	Normal	406.00	*CSF3R*(p.Q749*)19%; *ASXL2*(p.Q1037fs)2.2%; *JAK1*(p.V658F)16.7%; *ZBTB7A*(p.A175fs)18.6%
P076	M	69	2	9.00%	Adverse	0	1	1	Complex	No	Normal	Negative	*ASXL1*(p.I552fs)4.9%
P302	M	51	1	9.80%	Intermediate	0	0	0	Normal	No	Normal	Negative	*RTEL1*(p.M756fs)39.7%
